# Scale-up of the Internet-based Professional Learning to help teachers promote Activity in Youth (iPLAY) intervention: a hybrid type 3 implementation-effectiveness trial

**DOI:** 10.1186/s12966-022-01371-4

**Published:** 2022-12-01

**Authors:** D R Lubans, T Sanders, M Noetel, P Parker, H McKay, PJ Morgan, J Salmon, M Kirwan, A Bennie, L Peralta, R Cinelli, M Moodie, T Hartwig, J Boyer, S G Kennedy, R C Plotnikoff, V Hansen, D Vasconcellos, J Lee, D Antczak, C Lonsdale

**Affiliations:** 1 Centre for Active Living and Learning, College of Human and Social Futures, University of Newcastle, Callaghan, NSW Australia; 2 Hunter Medical Research Institute, New Lambton Heights, NSW Australia; 3 Faculty of Sport and Health Sciences, University of Jyväskylä, Jyväskylä, Finland; 4 Institute for Positive Psychology and Education, Australian Catholic University, North Sydney, NSW Australia; 5 School of Psychology, University of Queensland, Brisbane, QLD Australia; 6 Centre for Hip Health and Mobility, University of British Columbia, Vancouver, BC Canada; 7 Institute for Physical Activity and Nutrition (IPAN), School of Exercise and Nutrition Sciences, Deakin University, Geelong, VIC Australia; 8 Faculty of Medicine, Health and Human Sciences, Macquarie University, Macquarie Park, NSW Australia; 9 School of Health Sciences, Western Sydney University, Penrith, NSW Australia; 10 School of Education and Social Work, University of Sydney, Camperdown, NSW Australia; 11 School of Education, Australian Catholic University, Strathfield, NSW Australia; 12 Deakin Health Economics Deakin University, Burwood, VIC Australia; 13 School of Behavioural and Health Sciences, Australian Catholic University, Strathfield, NSW Australia; 14 NSW Department of Education, Turrella, NSW Australia; 15 Southern Cross University, East Lismore, NSW Australia; 16 North Sydney, Australia; 17 Global Centre for Modern Ageing, Tonsley, South Australia Australia

## Abstract

**Background:**

Whole-of-school programs have demonstrated success in improving student physical activity levels, but few have progressed beyond efficacy testing to implementation at-scale. The purpose of our study was to evaluate the scale-up of the ‘Internet-based Professional Learning to help teachers promote Activity in Youth’ (iPLAY) intervention in primary schools using the RE-AIM framework.

**Methods:**

We conducted a type 3 hybrid implementation-effectiveness study and collected data between April 2016 and June 2021, in New South Wales (NSW), Australia. RE-AIM was operationalised as: (i) Reach: Number and representativeness of students exposed to iPLAY; (ii) Effectiveness: Impact of iPLAY in a sub-sample of students (*n* = 5,959); (iii) Adoption: Number and representativeness of schools that received iPLAY; (iv) Implementation: Extent to which the three curricular and three non-curricular components of iPLAY were delivered as intended; (v) Maintenance: Extent to which iPLAY was sustained in schools. We conducted 43 semi-structured interviews with teachers (*n* = 14), leaders (*n* = 19), and principals (*n* = 10) from 18 schools (11 from urban and 7 from rural locations) to determine program maintenance.

**Results:**

Reach: iPLAY reached ~ 31,000 students from a variety of socio-economic strata (35% of students were in the bottom quartile, almost half in the middle two quartiles, and 20% in the top quartile). Effectiveness: We observed small positive intervention effects for enjoyment of PE/sport (0.12 units, 95% CI: 0.05 to 0.20, d = 0.17), perceptions of need support from teachers (0.26 units, 95% CI: 0.16 to 0.53, d = 0.40), physical activity participation (0.28 units, 95% CI: 0.10 to 0.47, d = 0.14), and subjective well-being (0.82 units, 95% CI: 0.32 to 1.32, d = 0.12) at 24-months. Adoption: 115 schools received iPLAY. Implementation: Most schools implemented the curricular (59%) and non-curricular (55%) strategies as intended. Maintenance: Based on our qualitative data, changes in teacher practices and school culture resulting from iPLAY were sustained.

**Conclusions:**

iPLAY had extensive reach and adoption in NSW primary schools. Most of the schools implemented iPLAY as intended and effectiveness data suggest the positive effects observed in our cluster RCT were sustained when the intervention was delivered at-scale.

**Trial registration:**

ACTRN12621001132831.

**Supplementary Information:**

The online version contains supplementary material available at 10.1186/s12966-022-01371-4.

## Background

The benefits of physical activity for young people are extensive [1], but physical inactivity is a global public health problem [2]. The Global Matrix of Physical Activity Report Grades was first launched in 2014 to provide a better understanding of the levels of youth physical activity across the world [3]. The most recent Global Matrix, with data from 49 countries, revealed an average grade of ‘D’. This indicates that only 20% to 40% of children and adolescents are sufficiently active for optimal health [3]. Schools are ideal settings to address low levels of physical activity, as they provide access to large and diverse groups of children, and typically have the resources, personnel and facilities to promote physical activity [4].

Whole-of-school programs (also known as Comprehensive School Physical Activity Programs) [5] are considered by the International Society for Physical Activity and Health to be one of ‘eight investments that work for physical activity’ [6]. Whole-of-school programs engage school communities to provide young people with multiple opportunities to be active throughout the day, including quality physical education (PE), active classrooms, active recess, and lunch breaks, after school activities, and the promotion of active transportation to-and-from school. Whole-of-school physical activity interventions are considered the ‘gold standard’ for increasing physical activity in youth [7], but few have been ‘scaled up’ to achieve maximum population impact [8, 9].

Scaling-up to expand the reach of efficacious health promoting interventions under real-world conditions into broader policy or practice is important, but challenging [10]. Of note, voltage drop (i.e., reduction in effectiveness) [11] typically occurs as interventions progress from efficacy to effectiveness to implementation at-scale [12, 13]. For example, Lane and colleagues found that scaled-up physical activity interventions achieve on average, less than 60% of their pre-scale effect size [13].

The Supporting Children’s Outcomes using Rewards, Exercise and Skills (SCORES) program [14, 15] was a whole-of-school physical activity intervention targeting children in low-income communities in New South Wales (NSW), Australia. The intervention successfully increased children’s objectively measured physical activity, cardiorespiratory fitness, and fundamental movement skill competency. However, SCORES relied heavily on support from researchers, thus limiting scalability. Guided by the Consolidated Framework for Implementation Research [16], we modified SCORES so that it could be delivered using an online platform with minimal in-person support from external mentors (i.e., experienced teachers employed by the project). The adapted intervention is known as iPLAY (internet-based Professional Learning to help teachers promote Activity in Youth) [17].

We evaluated iPLAY in a cluster randomised controlled trial (RCT) in 22 primary schools in New South Wales (NSW), Australia [18]. At 12- and 24-months, students in the iPLAY group had greater increases in cardiorespiratory fitness, compared with students in the control group. We also observed significant intervention effects for objectively measured physical activity during school lunch and recess breaks at 12- and 24-months, but no effects for total physical activity or other secondary outcomes. The cost of the intervention per student was AUD33 (USD26). Implementation-effectiveness of iPLAY was examined concurrently with the cluster RCT. Therefore, the aim of our current study was to evaluate implementation of iPLAY at broad scale in primary schools across New South Wales (NSW), Australia using the Reach, Effectiveness, Adoption, Implementation, and Maintenance (RE-AIM) framework [19]. Reach was considered the primary outcome in our implementation-effectiveness trial, as few school-based physical activity interventions progressed beyond smaller effectiveness to larger scale-up trials [8]. However, we acknowledge that all the components of the RE-AIM framework contribute to implementation success.

## Methods

### Study design

**Fig. 1:**
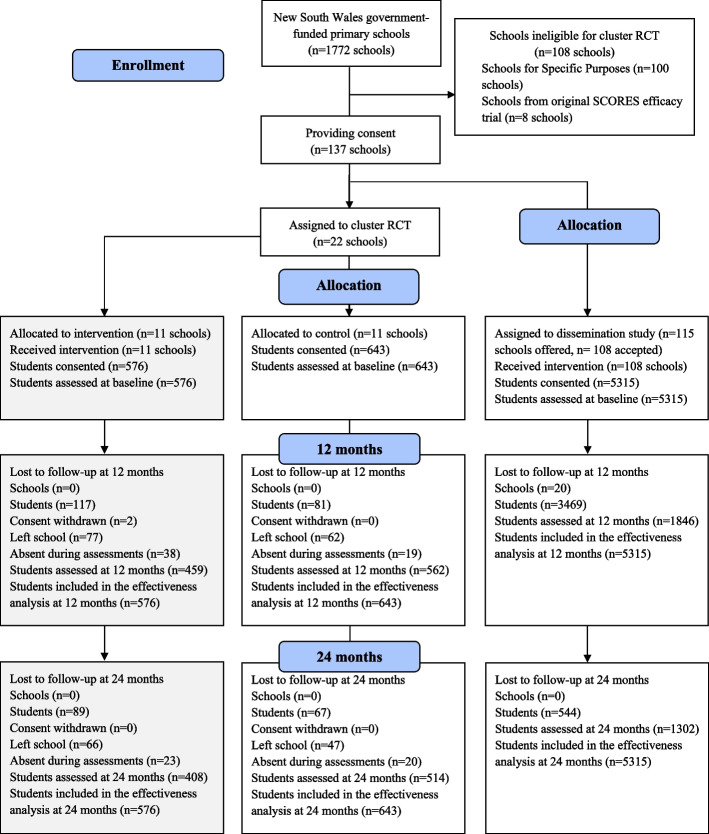
Adapted CONSORT flow diagram indicating participant flow throughout the study

We conducted a type 3 hybrid implementation-effectiveness study [20] in primary schools in NSW. The primary focus of a type 3 trial is to evaluate implementation strategies, whilst also evaluating effectiveness of the intervention at the individual-level. As such, program reach (i.e., estimated number and representativeness of students exposed to iPLAY) was considered the primary outcome of the study. The implementation-effectiveness study ran concurrently with the cluster RCT [18] which involved 22 schools. Control schools from the cluster RCT were compared with the sub-sample of schools that provided effectiveness data for the implementation-effectiveness study. Figure 1 provides an illustration of participants' flow through the cluster RCT and implementation-effectiveness studies. Approval for this study was provided by the Australian Catholic University (2014185) and University of Newcastle (H-2016–0135) human research ethics committees and the NSW Department of Education (DoE)(SERAP2014260). Our trial adheres to the Standards for Reporting Implementation Studies (StaRI) Statement [21] and was retrospectively registered with the Australian and New Zealand Clinical Trials Registry (ACTRN12621001132831).

### Participants and randomisation

All government-funded primary schools in NSW were considered eligible to participate (*N* = 1,808). Schools were recruited via presentations at conferences and meetings (e.g., regional meetings of the NSW Primary Principals Association) and advertisements sent by the NSW DoE and the Australian Council for Health, Physical Education and Recreation. The iPLAY study was also advertised via the NSW DoE Twitter feeds and Facebook pages. Recruitment of schools was on-going from 2016 to 2020. We aimed to recruit a total of 180 schools (~ 10% of the total number of NSW government-funded primary schools). We recruited 147 schools, of which 22 were assigned to the cluster RCT and 115 to the implementation-effectiveness study (8 schools did not start the program and 2 schools were involved in a pilot study). We used a blocked randomisation process to ensure that schools in the RCT broadly represented government schools in NSW (see protocol paper for further details) [17]. Principals and teachers provided written consent for this study. Study participants and their parents/caregivers were provided with information statements. Opt-out parental consent was applied, whilst students provided oral assent.

### Intervention

The iPLAY intervention has been described in detail in our protocol paper [17]. In summary, iPLAY is a whole-of-school physical activity intervention, that includes three curricular [(i) quality physical education [22], (ii) classroom energizers, and (iii) active homework], and three non-curricular [(iv) active playgrounds, (v) parental engagement, and (vi) community links] components. The program was designed to improve the primary outcome, cardiorespiratory fitness [23], by providing children with opportunities to participate in moderate-to-vigorous physical activity (MVPA) within and beyond the school setting.

### Implementation strategies

Implementation strategies comprised: (i) professional learning for teachers, (ii) access to the iPLAY website, and (iii) provision of support from iPLAY mentors. Specifically, teachers were trained to deliver iPLAY via a combination of face-to-face and online learning modalities. The training involved a 2-h face-to-face workshop, four hours of online learning (8 × 30-min modules), a mentoring meeting, a peer observation, and a discussion at a staff meeting focused on iPLAY implementation. Completing these activities provided each classroom teacher with 14 h of professional learning that was registered with the NSW Education Standards Authority (the government education authority with the responsibility for the establishment and monitoring of quality teaching, learning, assessment, and school standards in NSW).

School principals also chose 1–3 teachers to be school leaders. These individuals received further online training and were responsible for the implementation of the non-curricular iPLAY components. iPLAY was the first whole-of-school physical activity intervention where most teacher training occurred online, an implementation strategy chosen to support the scalability of iPLAY and enhance sustainability. Online delivery allowed teachers to complete their learning at a time that suited them. It also allowed program content to be standardised, ostensibly to limit voltage drop and prevent program drift [11]. The iPLAY website provided teachers and leaders with their online training modules, and access to downloadable resources (e.g., lesson plans, activity descriptions, and classroom movement break videos).

### Measures and outcomes

We used the RE-AIM framework [19] to guide the evaluation of iPLAY when implemented at-scale. The (RE-AIM) framework [19] was developed to address the slow translation of scientific knowledge into public health policy and practice [24]. The framework has been used extensively to guide scale-up of successful health promotion interventions [24] and allows researchers to assess internal and external validity. All dimensions of the RE-AIM framework are important, but we chose ‘Reach’ as our primary outcome because few school-based physical activity interventions have progressed beyond effectiveness trials. Data were collected at the individual (student, teacher, leader) and organisational (school principal) levels, via a combination of quantitative (questionnaires and website usage data) and qualitative (interviews with principals, leaders and teachers) methods. Table 1 provides a description of the different quantitative and qualitative methods used to assess the five RE-AIM dimensions. A summary of this information is provided below:

- *Reach* was defined as the estimated number and representativeness of students who were exposed to iPLAY. We accessed the MySchool website to obtain student enrolment data for schools that received iPLAY.
- *Effectiveness* was defined as the impact of the iPLAY program in a sub-sample of students from the implementation-effectiveness cohort (*n* = 5,315), who were compared with students from the control group in the cluster RCT (*n* = 643). The intervention designed to increase students’ physical activity within and beyond the school day. As such, we examined the impact of the intervention on students’ overall physical activity levels, active transportation to school, as well as their motivation and effort in PE and school sport. The intervention was guided by self-determination theory [12, 13], and we hypothesized that satisfying students’ basic psychological needs for competence, relatedness, and autonomy during PE and school sport would lead to improvements in well-being [14]. Students’ self-reported effort during PE/sport [25, 26], enjoyment during PE/sport [25], perceptions of needs support from teachers [27–29], typical physical activity participation [30], physical activity participation in the last week, organised sport participation (team and individual), active commuting to school [31], and subjective well-being (i.e., happiness and life satisfaction) [32].
- *Adoption* was defined as the total number and representativeness of schools and teachers that participated in iPLAY (using data from the MySchool website), as well as the proportion of teachers and leaders who completed the iPLAY training modules (using data from the iPLAY website).
- *Implementation* (fidelity) was defined as the extent to which the curricular and non-curricular components of the program were delivered as intended. Teachers were asked to self-report their implementation of the curricular and non-curricular components of the intervention using the iPLAY website. iPLAY mentors conducted observations of teachers’ PE lessons using the Supportive, Active, Autonomous, Fair and Enjoyable (SAAFE) framework [22]. Mentors assessed the quality of lesson delivery using 15 items aligned with the SAAFE principles (e.g., Teacher provided praise on student effort and improvement). Each item was scored on a 5-point Likert scale (1 = not at all true to 5 = very true) and the average of the 15 items was calculated and reported. Feedback from the lesson observations were uploaded into the iPLAY website by mentors.
- *Maintenance* was operationalised as the extent to which curricular and non-curricular iPLAY components were maintained in schools. We conducted 43 semi-structured interviews with teachers (*n* = 14), leaders (*n* = 19), and principals (*n* = 10) from 18 schools (11 from urban and 7 from rural locations) to determine program maintenance (see Supplementary Table 1 for the interview guide). Nine of the schools were classified as ‘high adopters’ (67 to 100% of online modules completed by teachers), six schools were classified as ‘medium adopters’ (34 to 66% of online modules completed by teachers), and three schools as ‘low adopters’ (0 to 33% of online modules competed by teachers). Interviews were completed 18- to 24-months from baseline.

**Table 1:** Operationalisation of RE-AIM dimensions, data sources, outcome detail and analysis

	**Reach**	**Effectiveness**	**Adoption**	**Implementation**	**Maintenance**
**Operationalisation**	The estimated number, proportion and representativeness of students who were exposed to the iPLAY program.	Impact of the iPLAY program on student outcomes.	The total number and representativeness of schools and teachers that participated in the iPLAY program.	The extent to which the curricular and non-curricular components of the program were delivered as intended.	The extent to which the iPLAY program has become institutionalised in schools.
			Proportion of teachers and leaders who completed iPLAY training modules.		
**Data sources**	iPLAY workshop enrolment and website	Questionnaires were completed by a sub-sample of students from the implementation-effectiveness cohort: baseline (*n* = 5,315), 12- (*n* = 1,846) and 24-months (*n* = 1,302). These questionnaires were also completed by control group students from the cluster RCT (*n* = 643)	iPLAY website	iPLAY website	Interviews with teachers (*n* = 20), leaders (*n* = 12) and principals (*n* = 10) completed ~ 18-months from baseline
	MySchool website (student enrolment data [by school] to evaluate implementation-effectiveness student characteristics)		Teacher demographics questionnaire (*n* = 1,359)	PE lesson observations (reported by iPLAY mentors)	
			MySchool website (school data, to evaluate school characteristics)		
**Outcome details**	School details were collected via workshop enrolment and also via the iPLAY website during teacher training. These details were then utilised in conjunction with the MySchool website, to assess student characteristics. Characteristics included: gender distribution, SES, Indigenous status, and language background other than English.	Students’ self-reported: enjoyment of PE/sport, perceptions of needs support from teachers, typical physical activity participation, physical activity participation in the last week, organised sport participation (team and individual), active commuting to school, and subjective well-being (i.e., happiness and life satisfaction).	Characteristics of schools in the implementation-effectiveness study were collected via the MySchool database, and included school size, SES, location, proportion of non-English speaking and Indigenous students.	**Curricular components** *Quantity of PE and school sport*- the number and proportion of teachers who reported at least 150 min of PE/school sport/week across all modules (i.e., up to 8 times, via website).	Extent to which iPLAY has become institutionalised in schools.
					Semi-structured interviews were conducted with 43 participants. This included 14 teachers, 19 leaders and 10 principals from 18 schools (7 rural and 11 urban schools. Nine of the schools were classified as ‘high adopters’, 6 schools as ‘medium adopters’, and 3 schools as ‘low adopters’.
				*Quality of PE and school sport*- the number and proportion of teachers who were rated > 3.0 /5 (average of 15 strategies) on the SAAFE evaluation checklist by mentors (i.e., at least 1 observation/teacher). Lessons were rated on 15 strategies that were aligned with the 5 SAAFE principles. Each strategy (e.g., teacher provided individual skill specific feedback) from 1 (*not all true*) to 5 (*very true*).	
			Characteristics of teachers were collected via survey prior to training, including: age, sex, years of teaching experience, and ethnicity.		
			Proportion of teachers who completed at least 50% of the 12 professional learning modules.		
				*Classroom movement breaks-* the number and proportion of teachers who provided > 10 activity breaks/week, self-reported at the start of each online learning module (via website).	
			Proportion of leaders who completed all 5 professional learning modules and attended at least one action plan meeting.		
				*Physically active homework-* the number and proportion of teachers who provided physically active homework once/week, self-reported at the start of each online learning module (via website).	
				**Non-curricular components** *Active playgrounds-* number and proportion of leaders reporting the implementation of active playground strategies (reported by mentors).	
				*Community physical activity links*- number and proportion of schools that utilised Sporting Schools funding (reported by principals). Number and proportion of schools that had at least one teacher gain accreditation with a recognized sporting organisation (reported by leaders)	
				*Parent and caregiver engagement-* number and proportion who: (i) distributed iPLAY parent newsletters, (ii) held an iPLAY parental information session, and (iii) organised 1 physically active school fundraiser event (reported by leaders).	

*Abbreviations:* *NSW* New South Wales, *PE* Physical education, *SAAFE* Supportive, Active, Autonomous, Fair, and Enjoyable, *SES* Socio-economic status

### Quantitative analysis

Descriptive statistics were used to calculate the primary outcome (Reach) using IBM SPSS version 28. Intervention effectiveness at the student level was determined by statisticians who were blinded to schools’ allocation using R version 3. We tested for between group differences in changes in students’ self-reported outcomes using mixed effects models with random effects for student, teacher, and school to account for clustering. Our analyses were consistent with the intention-to-treat principle [33] and included all participants randomized to treatment conditions, regardless of whether they completed follow-up assessments, using maximum likelihood to manage missing data. We compared students in the control group with those in the implementation-effectiveness trial. We ran mixed-effects models with a gaussian link function. We ran all models in R version 3 using Markov Chain Monte Carlo estimation. Results were considered not statistically significant if 95% CIs contained 0, and statistical tests were 2-tailed. We also report sub-group analyses for boys and girls, as we observed group-by-time interaction effects in our cluster RCT. Data were analysed from October to November 2021. Cohen’s *d* was calculated by dividing the mean difference in change by the standard deviation of change for each outcome.

### Qualitative analysis

Four members of the research team conducted 43 semi-structured interviews with intervention teachers, leaders, and principals to determine program maintenance. Interviews lasted between 11 and 36 min (average = 20 min) and were audio recorded before being de-identified and transcribed verbatim. A qualitative content analysis [34] was carried out using NVivo 12 to organise and store the data. Initially, a phase of data immersion took place. Due to the close alignment with the RE-AIM framework and the purpose of analysis, a content analysis with an unconstrained categorisation matrix was applied. The qualitative dataset was coded for correspondence with the pre-identified categories, with additional inductively derived categories created to capture all salient content. Once the entire qualitative dataset was coded, descriptive summaries were developed with the aim of conveying the meaning of the overarching categories using sub-categories and participant quotes.

## Results

### Reach

iPLAY reached ~ 31,000 students (115 schools), representing approximately 6% of the total NSW primary school student population (~ 500,000) [35]. Just over 50% of students were female; 25% of all students were from language backgrounds other than English (Supplementary Table 2). Almost 10% of students were of Aboriginal or Torres Strait Islander descent. Students were from a variety of SES strata, as assessed by socio-educational advantage quartiles; over 35% of students were in the bottom quartile, almost half in the middle two quartiles, and 20% in the top quartile for SES.

### Effectiveness

Characteristics and baseline values for outcome variables of students involved in the sub-sample to determine effectiveness are presented in Supplementary Table 3. This sub-sample (n = 5,959) included students from the implementation-effectiveness group (i.e., intervention group) and the control group (n = 643) from the cluster RCT. Students in the iPLAY implementation-effectiveness group reported improvements in a range of self-reported outcomes, compared with those in the control group (Table 2). Of note, there were small positive intervention effects for enjoyment of PE/sport (0.12 units, 95% CI: 0.05 to 0.20, *d* = 0.17), perceptions of need support from teachers (0.26 units, 95% CI: 0.16 to 0.53, *d* = 0.40), physical activity participation in the last week (0.28 units, 95% CI: 0.10 to 0.47, *d* = 0.14), and subjective well-being (0.82 units, 95% CI: 0.32 to 1.32, *d* = 0.12) at 24-months. Moderation effects by sex are reported in Supplementary Table 4.

**Table 2:** Effectiveness analyses for self-reported student outcomes

**Outcome**	**Follow-up** **(months)**	**N** **(n of Intervention)**	**Change from baseline: Control**	**Change from baseline: Intervention**	**Adjusted difference** **(Intervention vs Control)**^**a**^	**Effect size**^**b**^
Effort during PE/sport	12	2,359 (1802)	-0.07 (-0.13, -0.02)	-0.04 (-0.07, -0.01)	0.04 (-0.03, 0.10)	0.06 (-0.04, 0.16)
	24	1,780 (1274)	-0.24 (-0.29, -0.18)	-0.20 (-0.24, -0.17)	0.03 (-0.03, 0.10)	0.06 (-0.05, 0.16)
Enjoyment of PE/sport	12	2,359 (1802)	-0.14 (-0.20, -0.07)	-0.11 (-0.14, -0.07)	0.03 (-0.04, 0.10)	0.04 (-0.06, 0.14)
	24	1,780 (1274)	-0.33 (-0.40, -0.27)	-0.21 (-0.25, -0.17)	**0.12 (0.05, 0.20)**	0.17 (0.07, 0.28)
Perceptions of needs support from teachers	12	2,359 (1802)	-0.10 (-0.15, -0.04)	-0.04 (-0.07, -0.01)	0.06 (-0.00, 0.12)	0.08 (-0.00, 0.17)
	24	1,780 (1274)	-0.37 (-0.42, -0.31)	-0.07 (-0.11, -0.04)	**0.29 (0.22, 0.36)**	0.40 (0.31, 0.49)
Typical physical activity participation	12	2,370 (1813)	-0.06 (-0.22, 0.09)	0.20 (0.12, 0.28)	**0.26 (0.09, 0.44)**	0.13 (0.05, 0.22)
	24	1,782 (1276)	0.00 (-0.16, 0.16)	0.35 (0.25, 0.44)	**0.34 (0.16, 0.53)**	0.17 (0.08, 0.27)
Physical activity participation in last week	12	2,371 (1814)	0.04 (-0.11, 0.20)	0.23 (0.14, 0.31)	**0.18 (0.00, 0.36)**	0.09 (0.00, 0.18)
	24	1,778 (1272)	-0.05 (-0.21, 0.11)	0.24 (0.14, 0.33)	**0.28 (0.10, 0.47)**	0.14 (0.05, 0.24)
Organised team sport participation^c^	12	2,368 (1811)	-0.02 (-0.40, 0.35)	0.45 (0.24, 0.66)	**0.47 (0.04, 0.90)**	1.64 (1.07, 2.51)
	24	1,777 (1272)	0.39 (-0.01, 0.78)	0.18 (-0.06, 0.41)	-0.21 (-0.66, 0.25)	0.89 (0.56, 1.34)
Organised individual sport participation^c^	12	2,369 (1812)	-0.30 (-0.62, 0.01)	-0.50 (-0.66, -0.33)	-0.20 (-0.55, 0.16)	0.84 (0.59, 1.20)
	24	1,782 (1276)	-0.34 (-0.66, -0.01)	-0.54 (-0.73, -0.35)	-0.20 (-0.58, 0.18)	0.83 (0.57, 1.21)
Active commuting to school	12	2,366 (1809)	-0.11 (-0.26, 0.04)	0.03 (-0.06, 0.11)	0.14 (-0.04, 0.31)	0.06 (-0.02, 0.14)
	24	1,782 (1276)	0.22 (0.06, 0.38)	0.04 (-0.05, 0.14)	-0.17 (-0.36, 0.01)	-0.08 (-0.17, 0.01)
Subjective well-being	12	2,275 (1736)	0.10 (-0.31, 0.52)	0.34 (0.11, 0.56)	0.23 (-0.24, 0.70)	0.04 (-0.04, 0.12)
	24	1,718 (1221)	-0.88 (-1.30, -0.45)	-0.06 (-0.32, 0.21)	**0.82 (0.32, 1.32)**	0.14 (0.06, 0.23)

^a^Mixed effect models with a Poisson link function; bold results are statistically significant

^b^Cohen’s d = [(Intervention 24-month mean—Intervention baseline mean)—(Control 24-month mean—Control baseline mean)] / pooled standard deviation of change

^c^Binary outcome (log-odds) and odds ratio for effect size

### Adoption

A total of 115 schools were involved in the iPLAY implementation-effectiveness trial, which represents approximately 7% of all government-funded primary schools in NSW (Supplementary Table 5). Twenty schools dropped out of the study over the 4.5-year period. The implementation-effectiveness schools had a mean Index of Community Socio-educational Advantage (ISCEA) value of 990 and ranged from 732 to 1,182 (Australian median ISCEA value is 1,000). Most of the teachers were female, born in Australia; and ages ranged from 22–70 years (Supplementary Table 6).

Program adoption by school leaders is presented in Table 3. At 12-months, over 90% of leaders completed the five online learning modules, approximately 65% completed the four action plan meetings. At 24-months, the proportion of leaders completing online modules remained the same, whilst those completing action plans increased to 75%. Almost 90% of schools had at least one leader who completed all core learning, as per protocol, at both 12- and 24-months. Adoption of the intervention by teachers is also presented in Table 3. All teachers completed the mentor-facilitated professional learning workshop. At 12-months, almost 56% of teachers had completed all eight online learning modules, with this proportion increasing to 60% at 24-months. and 24-months.

**Table 3:** Intervention adoption rates by iPLAY leaders and teachers

**Core learning components**	**Proportion adoption**	
*Leader adoption*	**12 months**	**24 months**
Leader online learning (5 modules)	91%	91%
Leader action planning meetings (4 modules)	63%	75%
Leader adoption as per protocol^a^	87%	88%
*Teacher adoption*	**12 months**	**24 months**
Face-to-face teacher workshop (1 module)	100%	100%
Teacher online learning (8 modules)	56%	60%
Teacher school-based reflection (3 modules)	50%	50%
Teacher adoption as per protocol^b^	65%	67%

^a^At least one leader at the school completed all professional learning modules and attended at least one action plan meeting

^b^Teacher completed at least 50% of the 12 professional learning modules

### Implementation (fidelity)

Implementation fidelity data for the curricular and non-curricular iPLAY components are presented in Table 4 and Supplementary Fig. 1. Implementation of curricular components at 24-months is summarized here. *(i) Quality physical education:* Almost 60% of schools reported meeting the required 150 min of PE/sport per week. Mentor-rated quality of these lessons was moderate, with over 90% of teachers rated > 3.0/5 on the SAAFE evaluation checklist (see Table 1 for additional details). *(ii) Classroom energizers:* Almost half of teachers (48%) reported incorporating at least 10 classroom energizers per week. *(iii) Active homework:* Three quarters of teachers (75%) reported that they included one active homework activity each week. Non-curricular components were as follows. *(iv) Active playgrounds:* over half of leaders (53%) reported implementing strategies related to active playgrounds; however, no schools achieved > 40% of break time in MVPA. *(v) Parental engagement:* Just over 40% of schools distributed a newsletter to parents, with under one-third (30%) holding parent information sessions. Half of schools held a physically active school fundraiser. *(vi) Community links:* Principals reported that Sporting Schools (i.e., federal government program) funding was used by almost all schools, although less than 10% of schools used this funding to have a teacher complete an accredited sports coaching program*.*

**Table 4:** Curricular and non-curricular intervention implementation fidelity

	**Proportion implemented**	
**Teacher implementation—Curricular components**	**12-months**	**24-months**
150 min of PE/sport/week (teacher reported—median across modules)	59% teachers	59% teachers
Mean SAAFE rating > 3.0 rating (mentor-rated)^a^	91% teachers	92% teachers
≥ 10 classroom energizers per week (mean of teacher reports)	48% teachers	48% teachers
1 weekly active homework activity (mean of teacher reports)	75% teachers	75% teachers
Teacher implementation as per protocol^b^	57% teachers	59% teachers
**Leader Implementation—Non-curricular components**		**24-months**
Active playgrounds—Leader reports of implementing recommended strategies		53% schools
Sporting Schools funding used (principal report)		96% schools
At least one teacher complete accreditation to coach with a sporting body (teacher report)		9% schools
Parent newsletter distribution (Leader report)		41% schools
Parent info sessions (Leader report)		30% schools
One physically active school fundraiser (Leader report)		51% schools
Leader implementation as per protocol^c^		55% schools

^a^During observations, mentors rated teachers on the inclusion of the SAAFE principles within lessons

^b^Teacher implemented ≥ 50% of curricular strategies

^c^School implemented ≥ 50% of non-curricular strategies

### Maintenance

Findings from our interviews suggest being involved in iPLAY led to sustained changes in teacher practices and school culture. These changes meant that schools, leaders, and teachers placed a greater emphasis on whole school physical activity promotion, including quality PE and sport.

*We had a huge culture of literacy, numeracy which was really nice, but there was no real focus on sports. So since this program we've definitely made sure we allocated the right amount of hours properly. We've designed programs around the iPLAY resources from the website, which was really good. They are accessible to all teachers … they can access them whenever they want*. (Leader, Urban, High adoption)

Curricular features of iPLAY that teachers used in a sustained manner were; (i) reducing transition time, and (ii) maximising students’ opportunities to be active during lesson time. Similarly, teachers and leaders across all adoption levels revealed ongoing utilisation of active breaks and classroom energizers as a long-term legacy of their involvement in iPLAY.

*… from a personal level, I completely changed the way I teach sport, completely changed it. So from my warm ups, to my modified games, to that release of control, having students not just in student-centred games, but having the kids designed the game, giving the kids the freedom to practise the skills how they choose to and having that real focus on increasing physical activity within the lesson … not having that seat and explain time and having that explain-as-they-play kind of structure … We do a brain break Go Noodle thing, dance or activity twice a day in my classroom and the kids love it.* (Leader, Rural, High adoption)

Teachers also spoke favourably about sustaining the non-curricular elements of iPLAY following program delivery, such as changing the playground set-up to encourage varied physical activities in the school playground during recess and lunch times.

*I think that before iPLAY, you walked around the playground and it was handball. Whereas now you're seeing lots of games and activities and group work and things like that out on the playground.* (Teacher, Rural, Low adoption)

While many teachers spoke of immediate adoption and implementation of iPLAY curricular and non-curricular components, this was generally only a short-to-medium term change (i.e., 6 to 12 months). Enthusiasm waned once the iPLAY mentor presence and regular program engagement had ceased. For some schools, there was evidence that iPLAY resources were being used and sustainably integrated into school planning (high adoption schools), while for other schools this was less evident (low adoption schools).

*I probably would say that it's not so much iPLAY, the program itself or using iPLAY as a way to describe what we're doing. I think that's fallen away, that's not happening. But a lot of what we implemented when we were on iPLAY, so, the active playground stuff, the accessing old resources on the website using them to program PE and sport programs. That's still happening, but because they completed the program two years ago, we've had a high staff turnover, but also, people just think that's been in place, ‘cause it's been in place for a long time so we don't refer it to iPLAY anymore.* (Leader, Rural, High adoption)

Teachers from high and low adoption schools in rural and urban settings, highlighted the importance of iPLAY leaders and mentors to facilitate implementation of iPLAY. This was also evident in relation to the long-term maintenance of iPLAY, where leaders were seen as promoters of the program even after the study finished. Additionally, some teachers recommended maintaining longer-term connections with mentors to better sustain iPLAY program ideas.

*The leaders are still driving it. They're still asking too. ‘Can we do a professional learning session on this? I think we should focus on this fundamental movement skill’. Or whatever it may be … Maybe a little bit more training for the teachers [is needed] - the school-based mentors, so that they could kind of keep it perpetuating. And maybe giving them the time to be able to do that, some professional development that they could continue to do with their colleagues.* (Principal, Urban, Medium adoption)

In summary, there is evidence to suggest that iPLAY resulted in sustained changes in practices and culture. For many schools this included increased focus on PE and sport within programming, and the on-going implementation of non-curricular strategies such as classroom energiser breaks and provision of varied activities during recess and lunchtime periods. The provision of support (or lack thereof) from iPLAY leaders and mentors was identified as a key influence on long-term maintenance of iPLAY strategies.

## Discussion

As noted in the Lancet Physical Activity Series, few school-based physical activity interventions have progressed beyond effectiveness testing to be implemented at-scale [8]. This creates what has been termed a ‘know-do-dissemination gap’– that our study aimed to fill [36]. Bridging this gap is vital, as scaling up effective interventions is the only means to enhance the health of children and adolescents at a population level. Our trial represents one of the largest and most comprehensive type 3 hybrid implementation-effectiveness studies of a whole-of-school physical activity intervention published to date [9, 37, 38]. The iPLAY program reach more than 30,000 students and implementation fidelity was relatively high and consistent with what was observed in our cluster RCT. These findings hold great promise given the significant barriers to implementing school-based health promoting programs at-scale [39]. The World Health Organization [40] has identified two key reasons why implementation research continues to be neglected. These comprise a basic lack of knowing what implementation research is and its importance to health, and insufficient research funds to conduct implementation and scale-up studies, as costs often fall outside the capacity of most granting agencies.

The reach of iPLAY across five years was substantial– ~ 31,000 students from 115 primary schools in NSW, Australia (7% of all government schools). By comparison, reach ranged from 210 [41] to 1,000,000 [42] students in a recent systematic review of 14 school-based physical activity dissemination studies [9]. This variability is largely due to the different methods used to calculate reach in dissemination studies [9]. For example, previous studies have used the number of teachers attending professional learning workshops [43, 44] and the ordering of program materials by teachers, to estimate reach into the student population [45]. We defined ‘reach’ as the estimated number, proportion and representativeness of students who were potentially exposed to iPLAY. We estimated ‘reach’ at a single time point using student enrolment data accessed from the publicly available MySchool website. Our approach provides a conservative estimate of iPLAY’s reach because it does not include new students who enrolled in intervention schools each year with the now-trained teachers.

Our unique hybrid effectiveness type 3 study design allowed us to examine effectiveness of iPLAY on health outcomes in a sub-sample of students, who were compared with students in the control group from the cluster RCT [18]. Intervention effects for self-reported physical activity, participation in team sports, enjoyment of PE, teacher psychological need satisfaction in PE, and subjective well-being were observed at 24-months. This compares favourably with our cluster RCT [18], where we observed improvements in cardiorespiratory fitness (not measured in our implementation-effectiveness trial) and students’ perceived support from their teachers at 24-months, but no effects for other self-reported outcomes. However, we observed a trend toward improvements in well-being in our cluster RCT, which was confirmed in this larger study. We suggest that the large sample size included in our implementation-effectiveness study provided additional statistical power to detect small, but significant intervention effects. There is large variability in the way that effectiveness is calculated in dissemination studies, and student-level data are rarely collected [9]. For example, it is not uncommon for studies to refer to effectiveness data from a previous trial [44], and focus on implementation evaluation outcomes. Other studies have examined within group effects using a sub-study of participants from the larger dissemination study [43].

Of the 115 schools involved in our implementation-effectiveness trial, most were located in major cities, with almost a third from inner regional areas, and the remaining from outer regional areas. Our rate of adoption is lower than has been reported in previous school-based physical activity intervention dissemination studies [43, 46]. This may be attributed to a range of factors. First, rates of adoption have been measured in a variety of ways in previous studies. For instance, the Exercise Your Options (EYO) [46] and Resistance Training for Teens (RT for Teens) [43] programs were adopted by 42% and 46% of secondary schools in California and NSW, respectively. Adoption of the Exercise Your Options program was calculated as the proportion of middle school teachers who ordered the program materials. Similarly, adoption was based on the number and representativeness of schools with one or more teachers trained to deliver the RT for Teens program. Neither of these approaches reflect the level of commitment that was required in our implementation-effectiveness trial. Second, the high rates of adoption reported in the CATCH (since 1997) [45] and SPARK (since 1994) [42] programs are a direct reflection of the long period of time that these programs have been available to schools. Finally, iPLAY is a whole-of-school intervention that requires commitment from school principals and teachers. By comparison, programs such as RT for Teens, can be adopted by schools at the discretion of the Physical Education department.

We also examined the proportion of teachers and leaders who completed the iPLAY training modules as a measure of adoption. Similar to our RCT findings [18], 67% of our teachers completed at least 50% of the iPLAY professional learning modules (70% in the RCT). The typical completion rates for online professional learning are low, often due to low organisational support, insufficient provision of time, low perceived usefulness of content, and poor instructional design [47]. The high-levels of adoption observed in this implementation-effectiveness study may be attributable to our learning design choices that deliberately addressed these barriers (e.g., short online modules that fit in existing structures for professional learning opportunities, like team meetings). Leaders completing the core learning modules was also very high in our implementation-effectiveness trial (88%) and the RCT (100%). It is not surprising that these individuals were more motivated to complete the allocated training. They were more closely supported by iPLAY mentors and were accountable for supporting other teachers in their schools. Although rates of completion are higher for face-to-face professional learning opportunities, online training is more scalable [18]. Researchers and public health practitioners need to strike a balance between scalability and effectiveness. The implementation science literature is rife with discussion of the ‘adaptation-fidelity dilemma’ [10, 36, 48]. At scale, there must be a balance between the need to adapt an intervention to achieve best fit for a specific setting and delivery partner, while maintaining fidelity to the intervention as planned and delivered at smaller scale. Consistent with the iPLAY approach, we believe that online training modules should be supplemented with face-to-face support from external mentors, who provide support and accountability.

Consistent with the RE-AIM framework, we operationalised implementation at the ‘setting level’ and our key focus was teachers’ fidelity to delivering the six intervention components (i.e., quality physical education, (ii) classroom energizers, (iii) active homework], and three non-curricular [(iv) active playgrounds, (v) parental engagement, and (vi) community links) as intended. We demonstrated that iPLAY was delivered as intended in most schools (i.e., delivery of > 50% of curricular and non-curricular strategies). As interventions are delivered at increasing scale it creates a ‘dynamic tension’ between retaining fidelity to the intervention and adapting intervention components and program delivery to meet the needs of different delivery partners and different contexts [49]. At 24-months, 59% of teachers reported delivering at least 150 min of PE each school/week. Ninety-two percent (92%) of teachers’ PE lessons were rated ≥ 3.0 (out of 5) on the SAAFE evaluation checklist by our mentors. In addition, 48% of teachers reported delivering ≥ 10 classroom energizers/week and, and 75% doing one active home task/week. Schools’ implementation of the non-curricular intervention components ranged from 96% of schools applying for the Sporting Schools funding to 9% of schools having at least one teacher gain a coaching accreditation with a recognised sporting organisation. Rates of curricular and non-curricular implementation fidelity in our implementation-effectiveness trial were almost identical to those observed in our cluster RCT [18]. This is an important finding and provides evidence that our intervention design minimised ‘program drift’ and subsequent ‘voltage drop’ that typically occur as interventions progress from effectiveness to dissemination [11].

We conducted interviews with teachers, leaders, and principals to determine the extent to which iPLAY was maintained in schools. One of the most consistent points raised by interviewees was that iPLAY changed teacher practices and school culture. This was evidenced through a shift towards a greater emphasis on the programming of quality PE and sport. In most schools, there was clear evidence that iPLAY resources were still being used and integrated into school planning, particularly classroom energizer breaks after the first year of the intervention. Teachers found that energizer breaks were easy-to-implement and effective in helping students to focus in the classroom. It is perhaps not surprising that maintenance of iPLAY was greater in schools that had higher rates of adoption (i.e., higher completion rates of professional learning). This may be due to enhanced knowledge and skills acquired by teachers during professional development, and the associated feelings of confidence and competence to continue program delivery. Finally, principals noted the support (or lack of support) from leaders and mentors as being integral to influencing the long-term maintenance of iPLAY in schools.

### Limitations

It is important to note that our study was designed before the STaRI [21] and other guidelines for conducting implementation research were published [50, 51]. As such, there is now more guidance available for researchers conducting implementation research. Nevertheless, there are some study limitations that should be noted. First, our implementation-effectiveness trial was retrospectively registered, and we made some changes to our methods as the project progressed. For example, it seemed more appropriate to compare students in the implementation-effectiveness trial with those in the control group from the cluster RCT. We considered that an extant control group provided us the opportunity to conduct a more robust assessment of effectiveness. Our original approach involved examining within group changes. Second, most of the data used to evaluate reach, effectiveness, adoption, implementation, and maintenance were self-reported by participants. Nevertheless, these data were complemented by iPLAY mentors directly observing lessons, and from objective measures of website usage. Third, our effectiveness data were collected in a sub-sample of schools (~ 10%) and not all students who were assessed at baseline completed the 12- and 24-month assessments. Rates of drop-out are unlikely to have any effect on our findings, as mixed models are robust to missing data [33]. Finally, we did not specifically measure how teachers adapted the iPLAY intervention. Adaptation is deemed both inevitable and appropriate in dissemination studies [36], and would ideally be monitored and evaluated in future.

## Conclusions

Ours is one of very few whole-of-school physical activity interventions implemented at-broad scale that comprehensively assessed both effectiveness and implementation. We have demonstrated that iPLAY can be successfully scaled up using face-to-face and online learning, and support from an external mentor to reach more than 30,000 students. We also demonstrated that voltage drop is not inevitable when an intervention is implemented at scale, as teachers in 50% of schools were able to retain intervention fidelity. Importantly, positive changes in teacher practices and school culture were maintained over the longer term. We acknowledge that the cost of scaling-up school-based intervention studies are prohibitive in most cases, and require external funding support (e.g., from government). Studies that examine implementation strategies that minimise economic costs of scaling-up effective whole-of -school interventions, while retaining student level benefits are urgently needed.

## Supplementary Information

**Additional file 1: Supplementary Table 1.** iPLAY Teacher Interview Guide. **Supplementary Table 2.** Characteristics of students enrolled in implementation-effectiveness schools. **Supplementary Table 3.** Characteristics and baseline outcomes of students in the control and sub-sample of implementation-effectiveness schools. **Supplementary Table 4.** Effectiveness analyses for self-reported student outcomes by sex. **Supplementary Table 5.** Characteristics of schools enrolled in the implementation-effectiveness trial. **Supplementary Table 6.** Characteristics of teachers in the implementation-effectiveness schools.

**Additional file 2: Supplementary Figure 1.** Implementation of curricular and non-curricular intervention components.

## Data Availability

Study data and materials are not available publicly, however may be available upon request to the lead investigators (DRL and CL). All consenting participants were issued a unique identification number for confidentiality, and all data is stored securely as per ethical requirements.

## Abbreviations

CATCH: Child and Adolescent Trial for Cardiovascular Health
CRF: Cardiorespiratory fitness
DoE: Department of Education
iPLAY: Internet-based Professional Learning to help teachers to promote Activity in Youth
NSW: New South Wales
PE: Physical education
RCT: Randomised controlled trial
RE-AIM: Reach, effectiveness, adoption, implementation, maintenance
RT for Teens: Resistance Training for Teens
SAAFE: Supportive, Active, Autonomous, Fair, and Enjoyable
SES: Socio-economic status
SPARK: Sports, Play, and Active Recreation for Kids
US: United States
