# Supplementary material for: Scale-up of the Internet-based Professional Learning to help teachers promote Activity in Youth (iPLAY) intervention: a hybrid type 3 implementation-effectiveness trial
Source: Int J Behav Nutr Phys Act. 2022 Dec 1;19:141. doi: 10.1186/s12966-022-01371-4 (PMC9713961; doi:10.1186/s12966-022-01371-4)
Supplement: Supplementary file 1 — Additional file 1: Supplementary Table 1. iPLAY Teacher Interview Guide. Supplementary Table 2. Characteristics of students enrolled in implementation-effectiveness schools. Supplementary Table 3. Characteristics and baseline outcomes of students in the control and sub-sample of implementation-effectiveness schools. Supplementary Table 4. Effectiveness analyses for self-reported student outcomes by sex. Supplementary Table 5. Characteristics of schools enrolled in the implementation-effectiveness trial. Supplementary Table 6. Characteristics of teachers in the implementation-effectiveness schools. [file 12966_2022_1371_MOESM1_ESM.docx]

Supplementary Table 1: iPLAY Teacher Interview Guide

| **Principals** | **Leaders** | **Teachers** |
| --- | --- | --- |
| *Objective:* Determine the extent to which the iPLAY program has become institutionalised in schools. | *Objective:* Determine the extent to which the non-curricular iPLAY components have become institutionalised in schools. | *Objective:* Determine the extent to which the curricular iPLAY components have become institutionalised in schools. |
| **Extent to which iPLAY has been institutionalised** | | |
| Open ended: To what extent has iPLAY become part of your school’s culture?  Specific probes:   - Do teachers design and deliver their PE lessons using the SAAFE (Supportive, Active, Autonomous, Fair and Enjoyable) principles? - Does your school continue to provide opportunities for children to be physically active at recess and lunch-time? - Are teachers encouraged to use energiser breaks in the classroom? - Does your school have on-going links with community sport organisations? - Does your school continue to engage parents in physical activity promotion efforts? | Open ended: To what extent has iPLAY become part of your school’s culture?  Specific probes:   - Does your school continue to provide opportunities for children to be physically active at recess and lunch-time? - Are teachers encouraged to use energiser breaks in the classroom? - Does your school have on-going links with community sport organisations? Tell me about these. - Does your school continue to engage parents in physical activity promotion efforts? How? | Open ended: To what extent has iPLAY changed the way you teach PE and sport?  Specific probes  Do you remember the SAAFE principles? Can you remember what they are?  Do you use them in your PE and sport lessons?  Do you still use energiser breaks in the classroom? Why/why not?  Do you provide your students with active homework tasks? Why/why not? |

**Supplementary Table 2: Characteristics of students enrolled in implementation-effectiveness schools**

| **Characteristics** | **Implementation-Effectiveness Group**  **(n = 115 schools)** |
| --- | --- |
| Student enrolments  Total enrolments (N)  Female enrolments, (%)  Enrolments per school, mean (SD)  Female enrolments per school, mean (SD)  Male enrolments per school, mean (SD) | 31,080  16,166 (52%)  270.26 (229.78)  129.69 (113.21)  140.57 (117.42) |
| Language background other than English  Range, %  Mean (SD), % | 0-97  25.51 (28.39) |
| Aboriginal or Torres Strait Islander descent  Range, %  Mean (SD), % | 0-89  9.82 (12.96) |
| Socio-economic status, mean (SD) %; range^a^  Bottom quarter  Lower middle quarter  Upper middle quarter  Top quarter | 35.2 (24.06); 1-89  23.45 (8.02); 4-39  20.98 (9.63); 2-56  20.25 (20.72); 0-80 |

^a^ Socioeconomic data were not available for Schools for Specific Purposes (n =5).

**Supplementary Table 3.** **Characteristics and baseline outcomes of students in the control and sub-sample of implementation-effectiveness schools**

| **Outcome** | **RCT Control Group**  **(n= 643)** | **Sub-sample of Implementation-Effectiveness Group**  **(n= 5,315)** |
| --- | --- | --- |
| Age, mean (SD), y | 8.9 (0.7) | 8.7 (0.8) |
| Female participants, n (%) | 314 (49) | 2520 (48) |
| Born in Australia, n (%) | 571 (89) | 4689 (89) |
| English spoken at home, n (%) | 540 (84) | 4637 (88) |
| Aboriginal or Torres Strait Islander descent, n (%) | 58 (9.1) | 428 (8.7) |
| Index of Community Socio-Educational Advantage, mean (SD)^a^ | 1,014 (57) | 1,014 (65) |
| Remoteness index, n (%)  Rural  Urban | 107 (17)  536 (83) | 2,341 (44)  2,974 (56) |
| Concentration in lessons, mean (SD) | 4.48 (0.63) | 4.47 (0.60) |
| Effort during PE/sport, mean (SD) | 4.60 (0.60) | 4.44 (0.73) |
| Strategies employed when learning in PE/sport, mean (SD) | 3.95 (0.84) | 3.92 (0.87) |
| Enjoyment during PE/sport, mean (SD) | 4.54 (0.72) | 4.45 (0.80) |
| Perceptions of needs support from teachers, mean (SD) | 4.06 (0.73) | 3.96 (0.70) |
| Typical physical activity participation, days per week, n (%) |  |  |
| 0 | 6 (0.9%) | 196 (3.7%) |
| 1 | 34 (5.3%) | 378 (7.2%) |
| 2 | 48 (7.5%) | 583 (11%) |
| 3 | 70 (11%) | 572 (11%) |
|  |  |  |
| 4 | 70 (11%) | 616 (12%) |
| 5 | 94 (15%) | 667 (13%) |
| 6 | 89 (14%) | 655 (13%) |
| 7 | 226 (35%) | 1,568 (30%) |
| Physical activity participation in the last week, days per week, n (%) |  |  |
| 0 | 5 (0.8%) | 198 (3.8%) |
| 1 | 33 (5.2%) | 321 (6.1%) |
| 2 | 36 (5.6%) | 470 (9.0%) |
| 3 | 86 (13%) | 573 (11%) |
| 4 | 59 (9.2%) | 603 (11%) |
| 5 | 77 (12%) | 652 (12%) |
| 6 | 82 (13%) | 566 (11%) |
| 7 | 261 (41%) | 1,863 (36%) |
| Organised team sport participation, n (%) | 452 (71%) | 3,499 (67%) |
| Organised individual sport participation, n (%) | 417 (65%) | 3,145 (60%) |
| Active commuting to school, days per week, n (%) |  |  |
| 0 | 243 (38%) | 2,033 (39%) |
| 1 | 60 (9.4%) | 429 (8.2%) |
| 2 | 46 (7.2%) | 400 (7.6%) |
| 3 | 53 (8.3%) | 410 (7.8%) |
| 4 | 43 (6.7%) | 275 (5.2%) |
| 5 | 194 (30%) | 1,693 (32%) |
| Subjective well-being, mean (SD) | 40.9 (5.8) | 39.8 (5.9) |

^a^ Scale of socio-educational advantage computed for each school (ranges from 582 to 1,202, median = 1,003)

^b^ Australian Bureau of Statistics remoteness index by postcode. Dichotomous categorisation: urban (less remote) and rural (more remote).

**Supplementary Table 4: Effectiveness analyses for self-reported student outcomes by sex**

| **Outcome** | **Follow-up (months)** | **Sex** | **N**  **(n of Intervention)** | **Change from baseline: Control** | **Change from baseline: intervention** | **Adjusted difference**  **(Intervention vs Control)^a^** | **Effect size^b^** |
| --- | --- | --- | --- | --- | --- | --- | --- |
| Effort during PE/sport | 12 | boys | 1203 (919) | -0.10 (-0.17, -0.02) | -0.06 (-0.10, -0.02) | 0.037 (-0.05, 0.12) | 0.06 (-0.08, 0.20) |
| Effort during PE/sport | 24 | boys | 877 (622) | -0.20 (-0.28, -0.13) | -0.12 (-0.16, -0.07) | 0.089 (-0.00, 0.18) | 0.15 (-0.00, 0.30) |
| Effort during PE/sport | 12 | girls | 1127 (854) | -0.05 (-0.12, 0.03) | -0.08 (-0.19, 0.04) | -0.031 (-0.21, 0.14) | -0.05 (-0.34, 0.24) |
| Effort during PE/sport | 24 | girls | 885 (634) | -0.27 (-0.34, -0.19) | -0.23 (-0.35, -0.11) | 0.03 (-0.15, 0.21) | 0.05 (-0.25, 0.35) |
| Enjoyment of PE/sport | 12 | boys | 1203 (919) | -0.20 (-0.28, -0.11) | -0.14 (-0.18, -0.09) | 0.06 (-0.04, 0.16) | 0.08 (-0.05, 0.22) |
| Enjoyment of PE/sport | 24 | boys | 877 (622) | -0.29 (-0.38, -0.20) | -0.14 (-0.20, -0.09) | **0.14 (0.04, 0.25)** | 0.20 (0.05, 0.34) |
| Enjoyment of PE/sport | 12 | girls | 1127 (854) | -0.07 (-0.16, 0.02) | -0.20 (-0.33, -0.06) | -0.12 (-0.33, 0.08) | -0.17 (-0.45, 0.11) |
| Enjoyment of PE/sport | 24 | girls | 885 (634) | -0.38 (-0.47, -0.29) | -0.19 (-0.33, -0.05) | 0.20 (-0.01, 0.41) | 0.27 (-0.02, 0.56) |
| Perceptions of needs support from teachers | 12 | boys | 1203 (919) | -0.12 (-0.19, -0.04) | -0.05 (-0.09, -0.01) | 0.06 (-0.03, 0.15) | 0.09 (-0.03, 0.21) |
| Perceptions of needs support from teachers | 24 | boys | 877 (622) | -0.33 (-0.42, -0.25) | -0.04 (-0.09, 0.01) | **0.30 (0.20, 0.39)** | 0.41 (0.28, 0.54) |
| Perceptions of needs support from teachers | 12 | girls | 1127 (854) | -0.08 (-0.16, 0.00) | -0.05 (-0.17, 0.07) | 0.03 (-0.16, 0.21) | 0.04 (-0.22, 0.29) |
| Perceptions of needs support from teachers | 24 | girls | 885 (634) | -0.40 (-0.48, -0.32) | -0.05 (-0.17, 0.08) | **0.35 (0.16, 0.54)** | 0.48 (0.22, 0.75) |
| Typical physical activity participation | 12 | boys | 1215 (931) | -0.15 (-0.36, 0.07) | 0.20 (0.09, 0.32) | **0.35 (0.11, 0.59)** | 0.18 (0.05, 0.30) |
| Typical physical activity participation | 24 | boys | 877 (622) | 0.06 (-0.16, 0.29) | 0.37 (0.23, 0.51) | **0.31 (0.04, 0.57)** | 0.16 (0.02, 0.29) |
| Typical physical activity participation | 12 | girls | 1126 (853) | 0.03 (-0.19, 0.25) | 0.03 (-0.30, 0.36) | 0.00 (-0.50, 0.51) | 0.00 (-0.25, 0.26) |
| Typical physical activity participation | 24 | girls | 887 (636) | -0.06 (-0.28, 0.17) | 0.42 (0.07, 0.76) | 0.47 (-0.05, 1.00) | 0.24 (-0.03, 0.51) |
| Physical activity participation in last week | 12 | boys | 1215 (931) | 0.05 (-0.17, 0.27) | 0.33 (0.21, 0.44) | **0.28 (0.03, 0.52)** | 0.14 (0.02, 0.27) |
| Physical activity participation in last week | 24 | boys | 873 (618) | 0.03 (-0.20, 0.25) | 0.26 (0.12, 0.40) | 0.24 (-0.03, 0.50) | 0.12 (-0.02, 0.25) |
| Physical activity participation in last week | 12 | girls | 1127 (854) | 0.04 (-0.18, 0.26) | 0.12 (-0.22, 0.45) | 0.08 (-0.43, 0.59) | 0.039 (-0.22, 0.30) |
| Physical activity participation in last week | 24 | girls | 887 (636) | -0.12 (-0.35, 0.11) | 0.35 (-0.00, 0.70) | 0.47 (-0.06, 1.00) | 0.24 (-0.03, 0.50) |
| Organised team sport participation | 12 | boys | 1214 (930) | -0.28 (-0.83, 0.27) | 0.35 (0.05, 0.65) | 0.63 (-0.00, 1.26) | 1.871 (1.00, 3.51) |
| Organised team sport participation | 24 | boys | 872 (618) | 0.23 (-0.36, 0.83) | 0.03 (-0.31, 0.38) | -0.20 (-0.89, 0.49) | 0.82 (0.41, 1.62) |
| Organised team sport participation | 12 | girls | 1125 (852) | 0.32 (-0.19, 0.82) | 0.00 (-0.80, 0.81) | -0.31 (-1.50, 0.87) | 0.73 (0.22, 2.39) |
| Organised team sport participation | 24 | girls | 887 (636) | 0.63 (0.10, 1.16) | -0.15 (-1.00, 0.71) | -0.78 (-2.03, 0.47) | 0.46 (0.13, 1.60) |
| Organised individual sport participation | 12 | boys | 1214 (930) | -0.66 (-1.09, -0.24) | -0.54 (-0.76, -0.31) | 0.13 (-0.36, 0.61) | 1.14 (0.70, 1.84) |
| Organised individual sport participation | 24 | boys | 877 (622) | -0.49 (-0.93, -0.05) | -0.26 (-0.53, 0.01) | 0.23 (-0.28, 0.75) | 1.26 (0.75, 2.11) |
| Organised individual sport participation | 12 | girls | 1126 (853) | 0.11 (-0.35, 0.58) | -1.11 (-1.79, -0.43) | **-1.22 (-2.27, -0.16)** | 0.30 (0.10, 0.85) |
| Organised individual sport participation | 24 | girls | 887 (636) | -0.11 (-0.59, 0.36) | -1.17 (-1.9, -0.46) | -1.05 (-2.13, 0.03) | 0.35 (0.12, 1.03) |
| Active commuting to school | 12 | boys | 1214 (930) | -0.02 (-0.24, 0.19) | -0.04 (-0.15, 0.08) | -0.02 (-0.26, 0.23) | -0.01 (-0.12, 0.11) |
| Active commuting to school | 24 | boys | 877 (622) | 0.18 (-0.05, 0.40) | -0.04 (-0.18, 0.09) | -0.22 (-0.48, 0.04) | -0.10 (-0.23, 0.02) |
| Active commuting to school | 12 | girls | 1123 (850) | -0.20 (-0.42, 0.02) | 0.28 (-0.05, 0.61) | 0.49 (-0.02, 0.99) | 0.23 (-0.01, 0.46) |
| Active commuting to school | 24 | girls | 887 (636) | 0.25 (0.03, 0.48) | 0.11 (-0.24, 0.50) | -0.15 (-0.67, 0.37) | -0.07 (-0.31, 0.17) |
| Subjective well-being | 12 | boys | 1169 (892) | -0.08 (-0.65, 0.49) | 0.07 (-0.24, 0.38) | 0.15 (-0.50, 0.80) | 0.03 (-0.09, 0.14) |
| Subjective well-being | 24 | boys | 848 (597) | -1.01 (-1.60, -0.42) | 0.53 (0.15, 0.90) | **1.53 (0.83, 2.24)** | 0.26 (0.14, 0.38) |
| Subjective well-being | 12 | girls | 1078 (816) | 0.30 (-0.29, 0.89) | 0.29 (-0.60, 1.17) | -0.01 (-1.36, 1.34) | -0.00 (-0.23, 0.23) |
| Subjective well-being | 24 | girls | 852 (606) | -0.74 (-1.35, -0.14) | -0.84 (-1.76, 0.08) | -0.10 (-1.49, 1.30) | -0.02 (-0.26, 0.22) |

^a^Mixed effect models with a Poisson link function; bold results are statistically significant.

^b^Cohen’s d = [(Intervention 24-month mean - Intervention baseline mean) - (Control 24-month mean - Control baseline mean)] / pooled standard deviation of change.

**Supplementary Table 5: Characteristics of schools enrolled in the implementation-effectiveness trial**

| **Characteristics** | **Schools (n = 115)** |
| --- | --- |
| School year range, n (%)  K-12  K-6 | 1  114 |
| School location, n (%)^a^  Major Cities  Inner Regional  Outer Regional  Remote | 69 (60.0)  36 (31.3)  10 (8.7)  0 (0.0) |
| Index of Community Socio-Educational Advantage (ISCEA)^b^  Mean (SD)  Range | 990.22 (94.01)  732-1184 |
| Student enrolments  Total enrolments (N)  Female enrolments, (%)  Enrolments per school, mean (SD)  Female enrolments per school, mean (SD) | 31,080  16,166 (52%)  270.26 (229.78)  129.69 (113.21) |
| Language background other than English  Range, %  Mean (SD), % | 0-97  25.51 (28.39) |
| Aboriginal or Torres Strait Islander descent  Range, %  Mean (SD), % | 0-89  9.82 (12.96) |
| Socio-economic status, mean (SD) %; range^c^  Bottom quarter  Lower middle quarter  Upper middle quarter  Top quarter | 35.2 (24.06); 1-89  23.45 (8.02); 4-39  20.98 (9.63); 2-56  20.25 (20.72); 0-80 |

^a^ Remoteness classified by the Australian Bureau of Statistics, on the basis of a measure of relative access to services.

^b^ Scale of socio-educational advantage computed for each school (ranges from 582 to 1,202, median = 1,003).

^c^ Socioeconomic data were not available for Schools for Specific Purposes (n =5).

**Supplementary Table 6: Characteristics of teachers in the** i**mplementation-effectiveness schools**

| **Characteristics** | **Implementation-Effectiveness Group**  **(n=1,359)** |
| --- | --- |
| Age  Mean (SD), y  Range, y | 38.4 (10.7)  22-70 |
| Female participants, n (%) | 1,174 (86.7%) |
| Born in Australia, n (%) | 578 (91.2%) |
| Cultural background, n (%)  Australian  Europe  Africa  Asia  Oceania (excl Australia)  Americas | 578 (91.2%)  13 (2.1%)  8 (1.3%)  13 (2.1%)  10 (1.6%)  6 (0.9%) |
| Aboriginal or Torres Strait Islander descent, n (%) | 22 (3.5%) |
| Years of teaching experience | 11.5 (9.36) |
