# Supplementary material for: Scale-up of the Internet-based Professional Learning to help teachers promote Activity in Youth (iPLAY) intervention: a hybrid type 3 implementation-effectiveness trial
Source: Int J Behav Nutr Phys Act. 2022 Dec 1;19:141. doi: 10.1186/s12966-022-01371-4 (PMC9713961; doi:10.1186/s12966-022-01371-4)
Supplement: Supplementary file 2 — Additional file 2: Supplementary Figure 1. Implementation of curricular and non-curricular intervention components. [file 12966_2022_1371_MOESM2_ESM.docx]

**Supplementary Figure 1: Implementation of curricular and non-curricular intervention components**

**
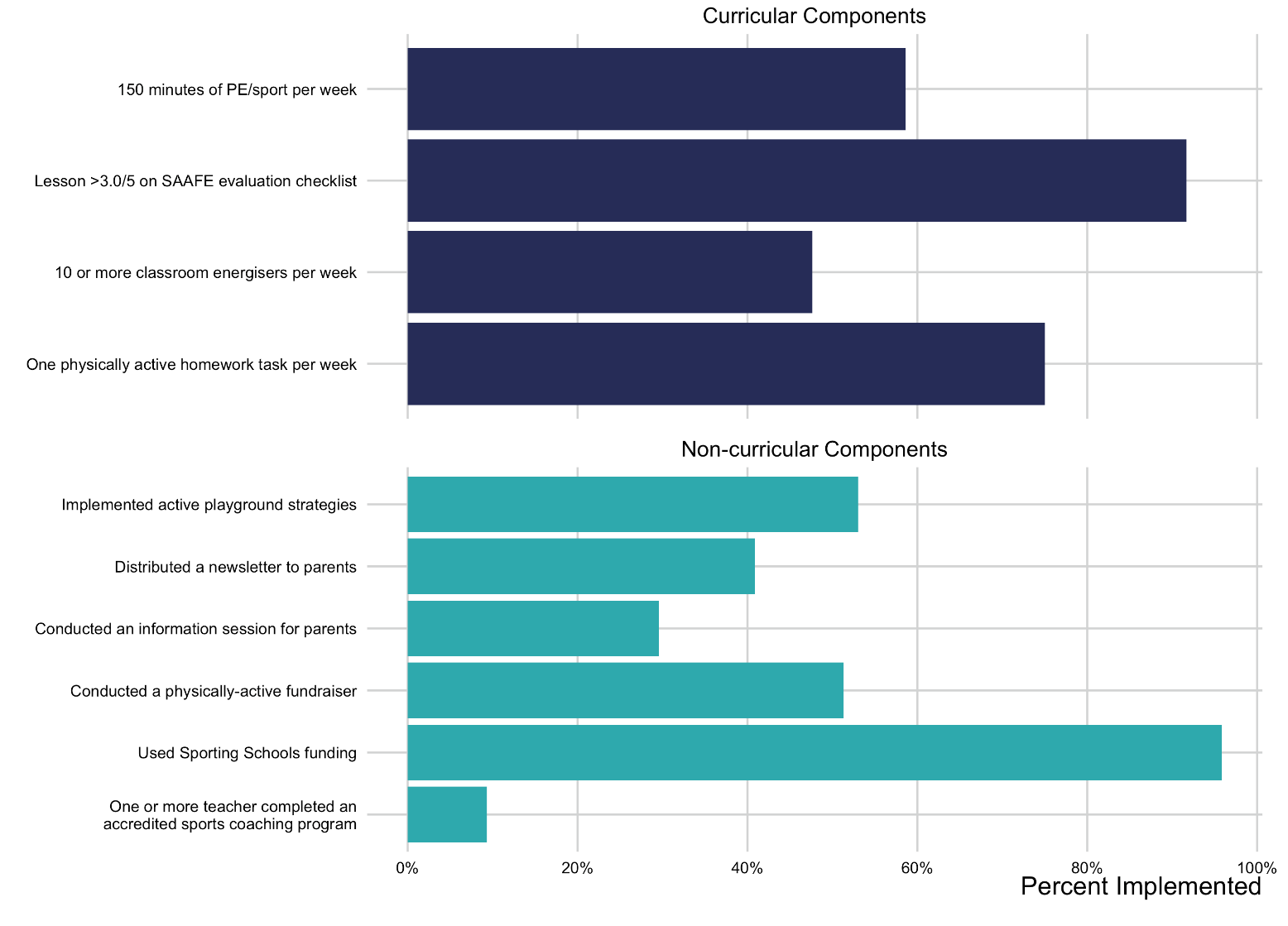
**
